# Keratinocyte transglutaminase 2 promotes CCR6^+^ γδT-cell recruitment by upregulating CCL20 in psoriatic inflammation

**DOI:** 10.1038/s41419-020-2495-z

**Published:** 2020-04-30

**Authors:** Ji-Woong Shin, Mee-ae Kwon, Jinha Hwang, Seok-Jin Lee, Jin-Haeng Lee, Hyo-Jun Kim, Ki Baek Lee, Soo-Jin Lee, Eui Man Jeong, Jin Ho Chung, In-Gyu Kim

**Affiliations:** 1 Department of Biochemistry and Molecular Biology, Seoul National University College of Medicine, Seoul, Republic of Korea; 2 Laboratory for Cellular Response to Oxidative stress, Cell2in, Inc., Seoul, Republic of Korea; 3 Department of Dermatology, Seoul National University College of Medicine, Seoul, Republic of Korea

**Keywords:** Transferases, Chemokines, Psoriasis

## Abstract

Keratinocyte-derived cytokines and chemokines amplify psoriatic inflammation by recruiting IL-17-producing CCR6^+^ γδT-cells and neutrophils. The expression of these cytokines and chemokines mainly depends on NF-κB activity; however, the pathway that activates NF-κB in response to triggering factors is poorly defined. Here, we show that transglutaminase 2 (TG2), previously reported to elicit a T_H_17 response by increasing IL-6 expression in a mouse model of lung fibrosis, mediates the upregulation of cytokines and chemokines by activating NF-κB in imiquimod (IMQ)-treated keratinocytes. TG2-deficient mice exhibited reduced psoriatic inflammation in skin treated with IMQ but showed systemic immune responses similar to wild-type mice. Experiments in bone marrow (BM) chimeric mice revealed that TG2 is responsible for promoting psoriatic inflammation in non-BM-derived cells. In keratinocytes, IMQ treatment activated TG2, which in turn activated NF-κB signaling, leading to the upregulation of IL-6, CCL20, and CXCL8 and increased leukocyte migration, in vitro. Consequently, TG2-deficient mice showed markedly decreased CCR6^+^ γδT-cell and neutrophil infiltration in IMQ-treated skin. Moreover, TG2 levels were higher in psoriatic skin than in normal skin and correlated with IL-6, CXCL8, and CCL20 levels. Therefore, these results indicate that keratinocyte TG2 acts as a critical mediator in the amplification of psoriatic inflammation.

## Introduction

Psoriasis is a chronic inflammatory skin disease characterized by dysregulated keratinocyte proliferation and differentiation and the infiltration of immune cells, such as dendritic cells (DCs), T-cells, and neutrophils in skin lesions^1^. Genetic analyses of psoriatic patients and anti-cytokine therapy evaluations reveal that the IL-23/IL-17 axis is critical for the activation of immune cells in the pathogenesis of psoriasis^2,3^. Studies in IL-23- or imiquimod (IMQ; a Toll-like receptor 7/8 agonist)-induced mouse models of psoriasis show that psoriatic inflammation is impaired in IL-22- and IL-17-deficient mice, thereby confirming the role of the IL-23/IL-17 axis^4,5^. In these mice, the majority of IL-17-producing cells in inflamed skin are TCRδ^+^ cells, whereas mice lacking γδT-cells exhibit impaired epidermal hyperplasia and IL-17 production, suggesting that γδT-cells are the major IL-17A, IL-17F, and IL-22 producers in psoriatic inflammatory skin lesions^6,7^. Moreover, γδT-cells exhibit chemotactic migration in response to CCL20 due to CCR6 receptors expressed on their surface^8^. Indeed, CCR6-deficient mice are highly resistant to IL-23- or IMQ-induced psoriatic inflammation due to the limited skin infiltration of IL-17-producing γδT-cells^9,10^. These studies highlight the importance of CCL20 in the development of psoriatic inflammation and its potential as a therapeutic target.

Increasing evidence indicates that keratinocytes have a role in the pathogenesis of psoriasis by secreting cytokines and chemokines^11,12^. In response to IL-17 and TNF-α, keratinocytes secrete NF-κB-dependent proinflammatory cytokines and chemokines, including IL-6, CXCL8, and CCL20^13,14^. IL-6 is a key factor in IL-23/T_H_17-mediated cutaneous inflammation, including psoriasis^15^, whereas CXCL8, a CXCR1 and CXCR2 ligand, promotes neutrophil recruitment to psoriatic lesions, leading to pustular formation^16^. CCL20, the unique CCR6 ligand, mediates skin infiltration of IL-17-producing γδT-cells and DCs. Notably, anti-CCL20-neutralizing antibodies or engineered CCL20 variants with minimal chemotactic activity prevent IL-17-producing γδT-cell infiltration in the skin of IL-23-injected mice, leading to IL-17 and IL-22 downregulation^9,17^. Because CCL20 is predominantly produced by keratinocytes in psoriatic lesions^18^, inhibiting CCL20 expression in keratinocytes could be a potential therapeutic strategy; however, the mechanisms regulating CCL20 expression in keratinocytes are poorly defined.

Transglutaminase 2 (TG2) is a calcium-dependent enzyme that mediates the post-translational modification of substrate proteins by catalyzing isopeptide-bond formation between glutamine and lysine or incorporating polyamines into glutamine residues, thereby regulating their activity^19^. TG2 is associated with the pathogenesis of inflammatory diseases, such as cancer, neurodegenerative diseases, fibrosis, and autoimmune diseases^20^. We previously reported that TG2 is essential for IL-6 production in lung epithelial cells, which amplify inflammation by stimulating T_H_17 differentiation in a mouse model of bleomycin-induced lung fibrosis^21^. Recently, we demonstrated that UV-B irradiation increases TG2 activity by mobilizing calcium in the endoplasmic reticulum, resulting in elevated inflammatory cytokine expression in keratinocytes and skin inflammation^22^ and suggesting that TG2 might be involved in inflammation. Interestingly, immunohistochemical analysis of skin biopsies from patients with psoriasis showed that TG2 is overexpressed in the basal epidermis of psoriatic skin lesions^23^ however, the causal relationships between TG2 expression and psoriasis remain unknown. Accordingly, in this study, we investigated the role of TG2 in the pathogenesis of psoriasis using an IMQ-induced mouse model of psoriatic skin inflammation.

## Results

### Reduced skin inflammation in a TG2-deficient mouse model of IMQ-induced psoriasis

**Fig. 1:**
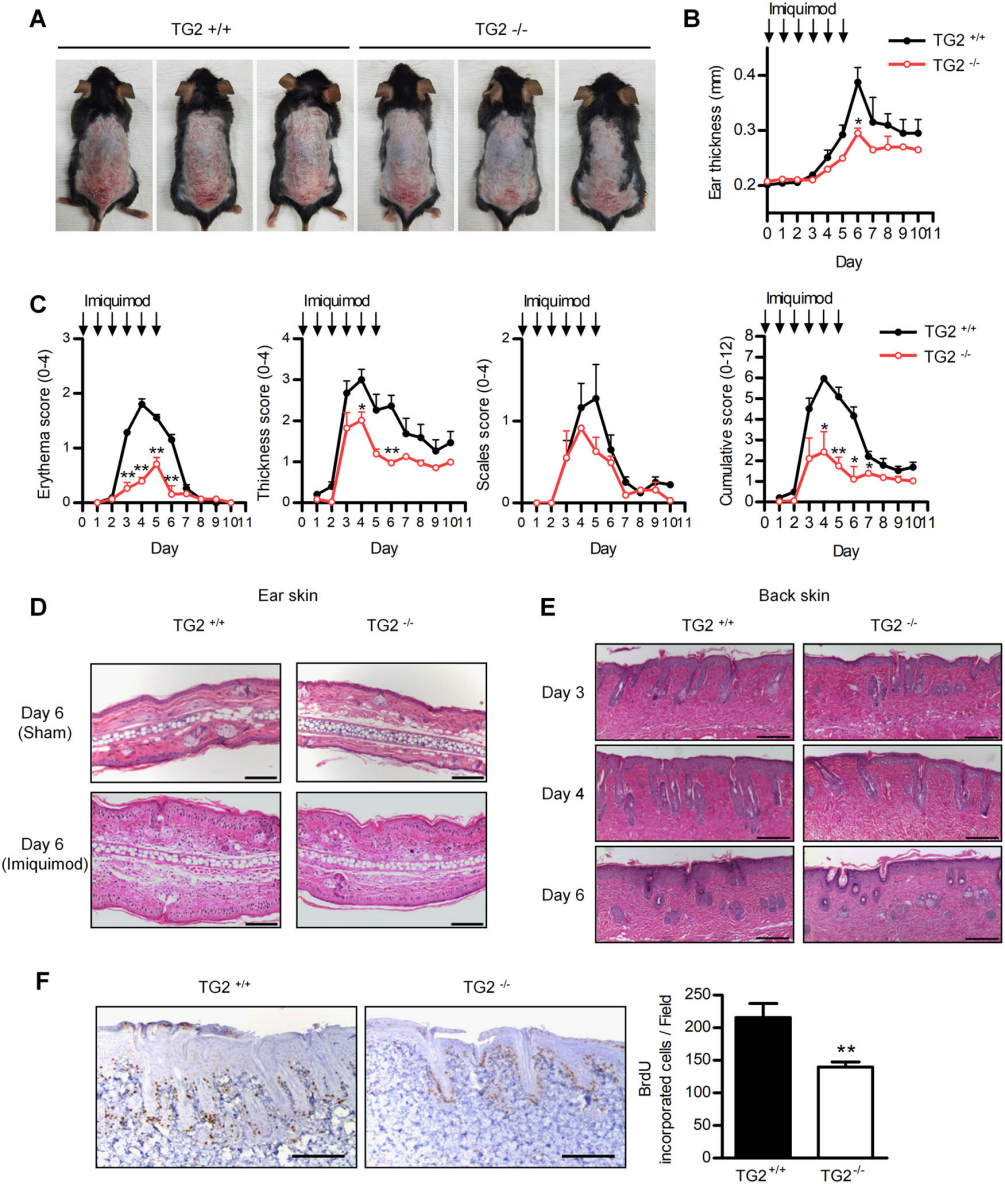
TG2 deficiency attenuates IMQ-induced psoriasis-like dermatitis. The right ear and shaved back skin of wild-type (WT) and TG2^*−/−*^ mice were treated with Aldara cream daily for 6 days, and skin inflammation was evaluated. **a** Phenotypic representation of psoriasiform lesions in WT and TG2^*−/−*^ mice on day 4. **b** Ear-skin thickness of WT and TG2^*−/−*^ mice measured daily for 10 days (*n* = 8/group). **c** Back skin erythema, thickness, scaling, and cumulative score assessed daily (scale: 1–10) (*n* = 8/group). **d**, **e** Ear (**d**) and back skin (**e**) H&E staining for WT and TG2^*−/−*^ mice. **f** BrdU incorporation was detected by immunohistochemistry (*left*). Bars (*right*) represent the mean number of BrdU-positive cells (*n* = 8/group). Data represent the mean ± SEM. *p < 0.05; **p < 0.01 vs. WT mice.

To determine whether TG2 is implicated in psoriasis pathogenesis, IMQ-containing Aldara cream was applied topically to the shaved back skin and right ear of wild-type (WT) and TG2^*−/−*^ mice for six consecutive days, as described previously^24^. The development of IMQ-induced psoriatic dermatitis was evaluated by measuring ear thickness and scoring the psoriasis area and severity index (PASI) for 10 days from the start of treatment. Erythema and scaling were reduced in TG2^*−/−*^ mice as compared with WT mice on day 4 of Aldara treatment (Fig. 1a), with TG2^*−/−*^ mice showing a significant reduction in ear thickness on day 6 (Fig. 1b) and a markedly lower PASI score relative to WT mice (Fig. 1c). Histologic examination of H&E-stained ear sections from IMQ-treated mice revealed that TG2^*−/−*^ mice showed reduced epidermal thickness on day 6 after treatment (Fig. 1d). In addition, H&E-stained dorsal skin sections from these mice demonstrated that IMQ-induced acanthosis was attenuated in TG2^*−/−*^ mice on days 3, 4, and 6 as compared with WT mice (Fig. 1e). Furthermore, in vivo BrdU-incorporation assays confirmed that hyperkeratosis was reduced in TG2^*−/−*^ mice, which displayed fewer BrdU-positive cells in the basal cell layer than WT mice (Fig. 1f). These findings indicate that TG2 is involved in promoting skin inflammation in IMQ-treated mice.

### TG2 in non-immune cells is responsible for promoting skin inflammation

To investigate how TG2 promotes IMQ-induced skin inflammation, we assessed whether TG2 activates immune cells. Cells were isolated from the lymph nodes (LNs) and spleens of IMQ-treated WT and TG2^*−/−*^ mice every other day for 10 days and analyzed by flow cytometry using immune cell-specific markers. Increased T_H_1, T_H_2, T_H_17, and T_reg_ cell percentages were observed in both the LN and spleen, peaking on day 4; however, there were no differences in immune-cell populations between the WT and TG2^*−/−*^ mice (Supplementary Fig. S1a, b).

We then examined whether TG2 affected IMQ-induced DC maturation. Bone marrow (BM) cells from WT and TG2^*−/−*^ mice were differentiated into immature DCs using GM-CSF and IL-4, and maturation was induced by treatment with various IMQ concentrations. In addition, DC maturation was determined by the percentage of CD80 and CD86 or MHC class II and CD40 double-positive cells using flow cytometry. We found that TG2 showed no observable effect on IMQ-induced DC maturation, despite increased TG enzyme activity in an IMQ-dose-dependent manner (Supplementary Fig. S2a, b). Moreover, our previously reported data showed that T-cell-expressed TG2 is not involved in T_H_17 and T_reg_ differentiation in vitro^21^; therefore, these data indicate that TG2-mediated improvements in IMQ-induced inflammation are not associated with systemic immune-cell activation.

**Fig. 2:**
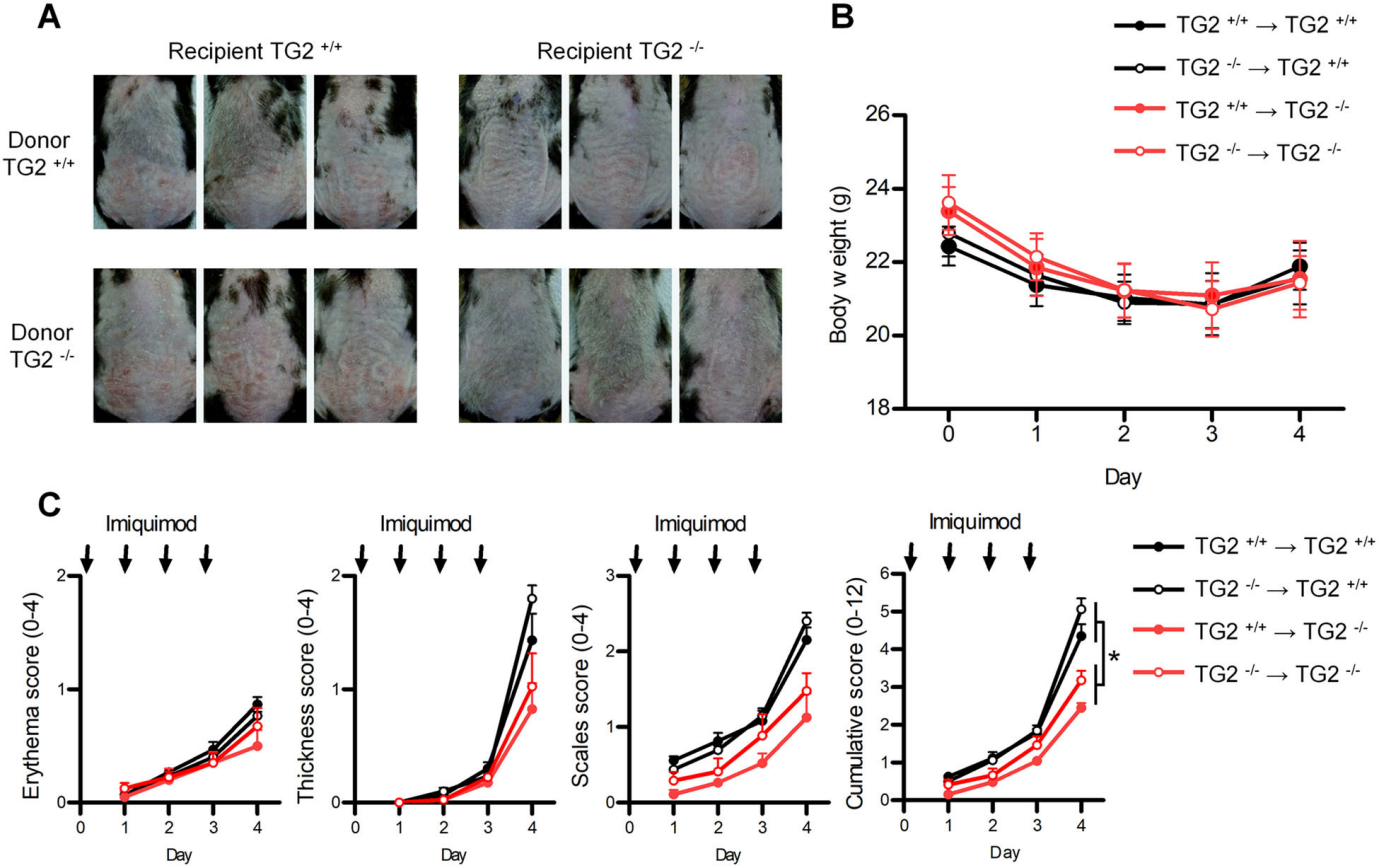
TG2 in non-BM-derived cells is involved in the development of IMQ-induced psoriasiform dermatitis. BM-chimeric mice were prepared by irradiating WT or TG2^*−/−*^ mice, followed by BM-cell reconstitution [BM^WT^ → WT (*n* = 6), BM^TG2*−/−*^ → WT (*n* = 5), BM^WT^ → TG2^*−/−*^ (*n* = 8), and BM^TG2*−/−*^ → TG2^*−/−*^ (*n* = 8)]. BM-chimeric mice were treated with IMQ for four consecutive days. **a** Phenotypic representation of psoriasiform skin lesions of chimeric mice. **b** The total body weight of mice was measured daily. **c** Back skin erythema, scaling, thickness, and cumulative score of IMQ-treated BM-chimeric mice on the indicated days. Data represent the mean ± SEM. *p < 0.05; **p < 0.01.

To confirm these findings, we created four chimeric mouse combinations by BM transplantation in WT and TG2^*−/−*^ mice and topically applied Aldara cream. Body weight did not differ between groups during the experimental period, indicating that TG2 deficiency did not affect the establishment of BM-chimeric mice (Fig. 2b). Conversely, TG2-deficient recipient mice displayed less macroscopic inflammation than WT recipient mice 4 days after treatment, regardless of donor BM-cell TG2 expression (Fig. 2a). Furthermore, TG2-deficient recipient chimeric mice displayed lower PASI scores (Fig. 2c). Collectively, these data indicate that TG2 expressed in non-immune cells has a role in promoting IMQ-induced psoriasiform dermatitis.

### TG2 mediates IMQ-induced IL-6, CXCL8, and CCL20 expression in keratinocytes

**Fig. 3:**
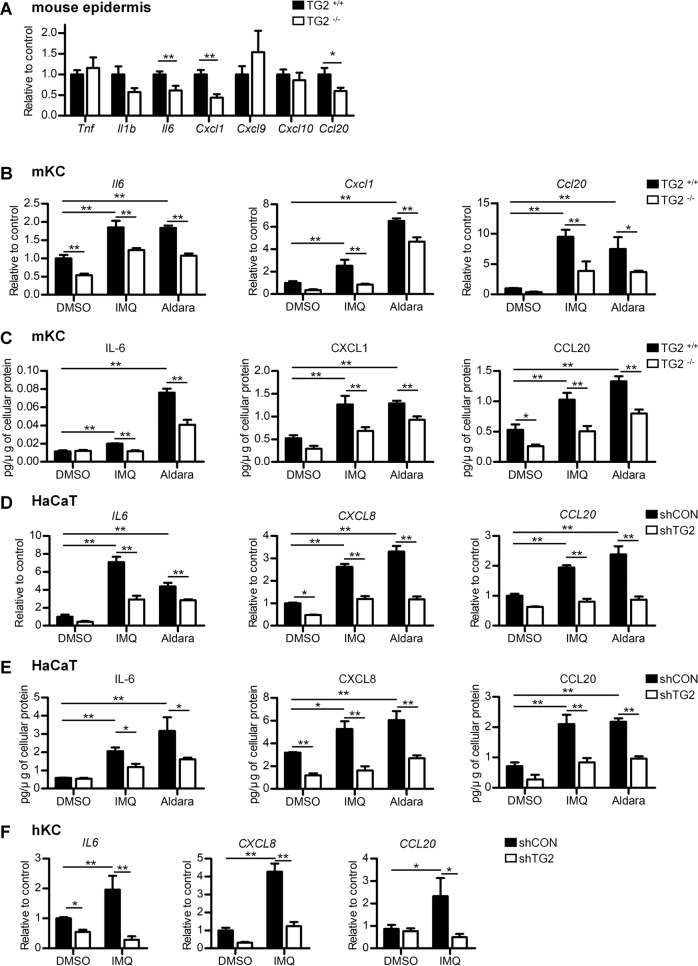
TG2 mediates IMQ-induced IL-6, CXCL8, and CCL20 expression in keratinocytes. **a** WT and TG2^*−/−*^ mice (*n* = 11/group) were treated with IMQ for four consecutive days. Cytokine and chemokine mRNA levels in the back skin epidermis were measured by RT-PCR. **b**, **c** Primary keratinocytes prepared from WT and TG2^*−/−*^ mice were treated with IMQ (200 μM) or Aldara cream (25 μM) for 3 h. *Ccl20*, *Il6*, and *Cxcl1* mRNA (**b**) and protein levels in the media (**c**) were measured by RT-PCR and a multiplex cytometric bead array, respectively (*n* = 3/group). **d**, **e** HaCaT cells stably transfected with control and TG2 shRNA were treated with IMQ (200 μM) or Aldara cream (25 μM). *IL6*, *CXCL8*, and *CCL20* mRNA (**d**) and protein levels in the media (**e**) were measured by RT-PCR after 3 h and a multiplex cytometric bead array after 6 h, respectively. Protein levels were normalized to total cellular soluble protein (*n* = 3/group). **f** Human primary keratinocytes stably transfected with control or TG2 shRNA were treated with IMQ (200 μM; *n* = 3). *IL6*, *CXCL8*, and *CCL20* mRNA levels were measured by RT-PCR after 3 h. Data represent the mean ± SEM (*n* = 3/group). *p < 0.05; **p < 0.01.

Because IMQ induces cytokine and chemokine expression in mouse and human keratinocytes that contribute to psoriatic inflammation^25,26^, we investigated whether TG2 is involved in IMQ-induced cytokine expression in keratinocytes by preparing WT and TG2^*−/−*^ mouse epidermis after 4 days of Aldara application and measuring psoriatic cytokine mRNA levels. *Il6*, *Cxcl1*, and *Ccl20* but not *Tnf*, *Il1b*, *Cxcl9*, and *Cxcl10* mRNA levels were significantly lower in TG2^*−/−*^ epidermis relative to WT epidermis (Fig. 3a). We confirmed these results using primary epidermal keratinocytes (mKCs) from WT and TG2^*−/−*^ mice. IMQ treatment increased *Il6*, *Cxcl1*, and *Ccl20* mRNA (Fig. 3b) and secreted protein (Fig. 3c) levels in WT-mKCs, whereas this effect was abolished in TG2^*−/−*^ mKCs. Similarly, when we examined cytokine production in human keratinocytes using shTG2-HaCaT cells, IMQ treatment significantly increased *IL6*, *CXCL8* (orthologue of mouse *Cxcl1*), and *CCL20* mRNA (Fig. 3d) and secreted protein levels in the media (Fig. 3e), which were reduced in TG2-downregulated HaCaT cells. These results were confirmed in human primary keratinocytes (hKCs), with IMQ increasing *IL6*, *CXCL8*, and *CCL20* mRNA levels; however, this effect was abolished in shTG2-hKCs (Fig. 3f). In addition, IMQ treatment did not increase mRNA levels of *TNF*, whereas *IL1B* level was increased in an IMQ-dose-dependent manner 3 h after treatment in both shCON- and shTG2-HaCaT cells (Supplementary Fig. S3a). However, secreted TNF-α and IL-1β levels in the media were not elevated following IMQ treatment (Supplementary Fig. S3b), and mRNA levels of *CXCL9* and *CXCL10*, which were related to the T_H_1 response, were not elevated following IMQ treatment, regardless of TG2 status in HaCaT cells (Supplementary Fig. S3c).

In psoriasis pathogenesis, T-cell-derived interferon (IFN)-γ, IL-17A, and TNF-α stimulate keratinocytes to produce proinflammatory cytokines and chemokines^13,27^; therefore, we determined whether TG2 is implicated in TNF-α-, IFN-γ-, and IL-17A-induced cytokine production in keratinocytes. TNF-α upregulated mRNA levels of all psoriatic cytokines and chemokines measured in this experiment, with *IL6* and *CCL20* levels significantly lower in shTG2-HaCaT cells as compared with levels in control cells (Supplementary Fig. S4a). IFN-γ increases *IL6*, *CXCL9*, and *CXCL10* mRNA levels; however, we found that *IL6* mRNA levels decreased in shTG2-HaCaT cells, whereas *CXCL9* and *CXCL10* levels increased (Supplementary Fig. S4b). Notably, IL-17A had a similar effect on cytokine expression as IMQ treatment, increasing *IL6*, *CXCL8*, and *CCL20* mRNA levels, which were reduced in shTG2-HaCaT cells (Supplementary Fig. S4c). Collectively, these results indicate that TG2 critically regulates IMQ or inflammatory cytokine-induced IL-6, CXCL8, and CCL20 expression in keratinocytes.

### IMQ activates TG2 in keratinocytes, leading to CCL20 expression by activating NF-κB

**Fig. 4:**
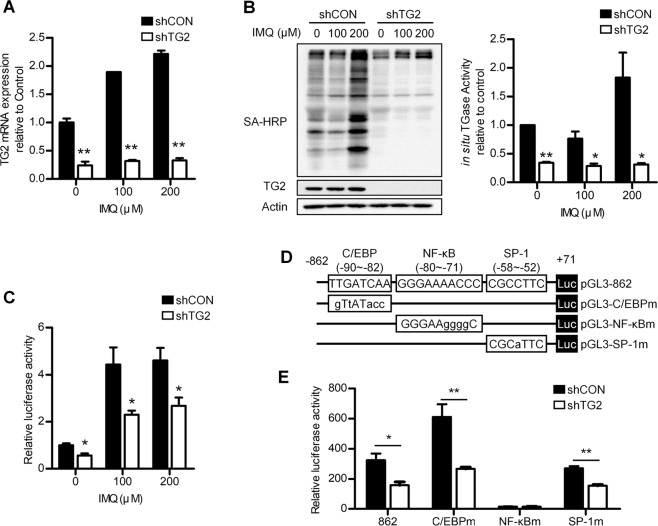
IMQ activates TG2 in keratinocytes, leading to *CCL20* expression by activating NF-κB. **a** HaCaT cells stably transfected with control and TG2 shRNA were treated with IMQ, and TG2 mRNA levels were determined by RT-PCR. **b** HaCaT cells were treated with IMQ in the presence of 5-(biotinamido) pentylamine (BP), and their lysates were subjected to western blot analysis with peroxidase-conjugated streptavidin to detect BP-incorporated proteins. In situ TG activity was measured according to band intensity by ImageJ software (*n* = 3/group). **c** HaCaT cells co-transfected with NF-κB-Luc reporters and phRL-TK control vectors were treated with IMQ for 6 h. NF-κB activity was represented by dual luciferase activity normalized to the control (*n* = 3/group). **d** Schematic representation of WT and mutated CCL20-promoter reporter plasmids. Transcription factor-binding sites are indicated. **e** HaCaT cells co-transfected with reporter constructs and phRL-TK control vectors for 48 h were treated with 200 μM IMQ for 12 h. CCL20-promoter activity was estimated by normalizing firefly luciferase luminescence to phRL-TK vector Renilla luciferase. Data are relative to the pGL3 control (*n* = 4/group) and represent the mean ± SEM. *p < 0.05; **p < 0.01.

To investigate the mechanism by which TG2 regulates CCL20 expression in IMQ-treated keratinocytes, we first examined whether IMQ influences TG2 activity. In HaCaT cells, IMQ treatment dose-dependently upregulated TG2 mRNA levels (Fig. 4a) and increased in situ TG activity in shCON- but not shTG2-HaCaT cells after 3 h (Fig. 4b). TG2 activates NF-κB signaling in response to various types of oxidative stress^21,22,28^, whereas IMQ induces cytokine expression by activating NF-κB^25,26^. Therefore, we tested whether TG2 is responsible for NF-κB activation in IMQ-treated keratinocytes. In HaCaT cells transfected with an NF-κB-Luc reporter construct, IMQ treatment increased luciferase activity in shCON- but to a lesser extent in shTG2-HaCaT cells (Fig. 4c), indicating that TG2 mediates IMQ-induced NF-κB activation in keratinocytes.

We confirmed these results using a well-characterized *CCL20*-promoter containing C/EBP, NF-κB, and SP-1 transcription factor-binding sites^29^. HaCaT cells were transfected with *CCL20*-promoter-Luc reporter constructs and treated with IMQ (Fig. 4d). NF-κB-binding-site mutations significantly decreased IMQ-induced luciferase activity as compared with that in the WT construct, whereas C/EBP- or SP-1-binding-site mutations had no effect. Conversely, IMQ treatment significantly reduced luciferase activity in transfected TG2-knockdown HaCaT cells, regardless of C/EBP- or SP-1-binding-site mutations (Fig. 4e). These results demonstrate that TG2 positively regulates IMQ-induced CCL20 transcription by activating NF-κB signaling.

### TG2 promotes CCR6^+^ γδT-cell infiltration in IMQ-treated skin by increasing CCL20 secretion in keratinocytes

**Fig. 5:**
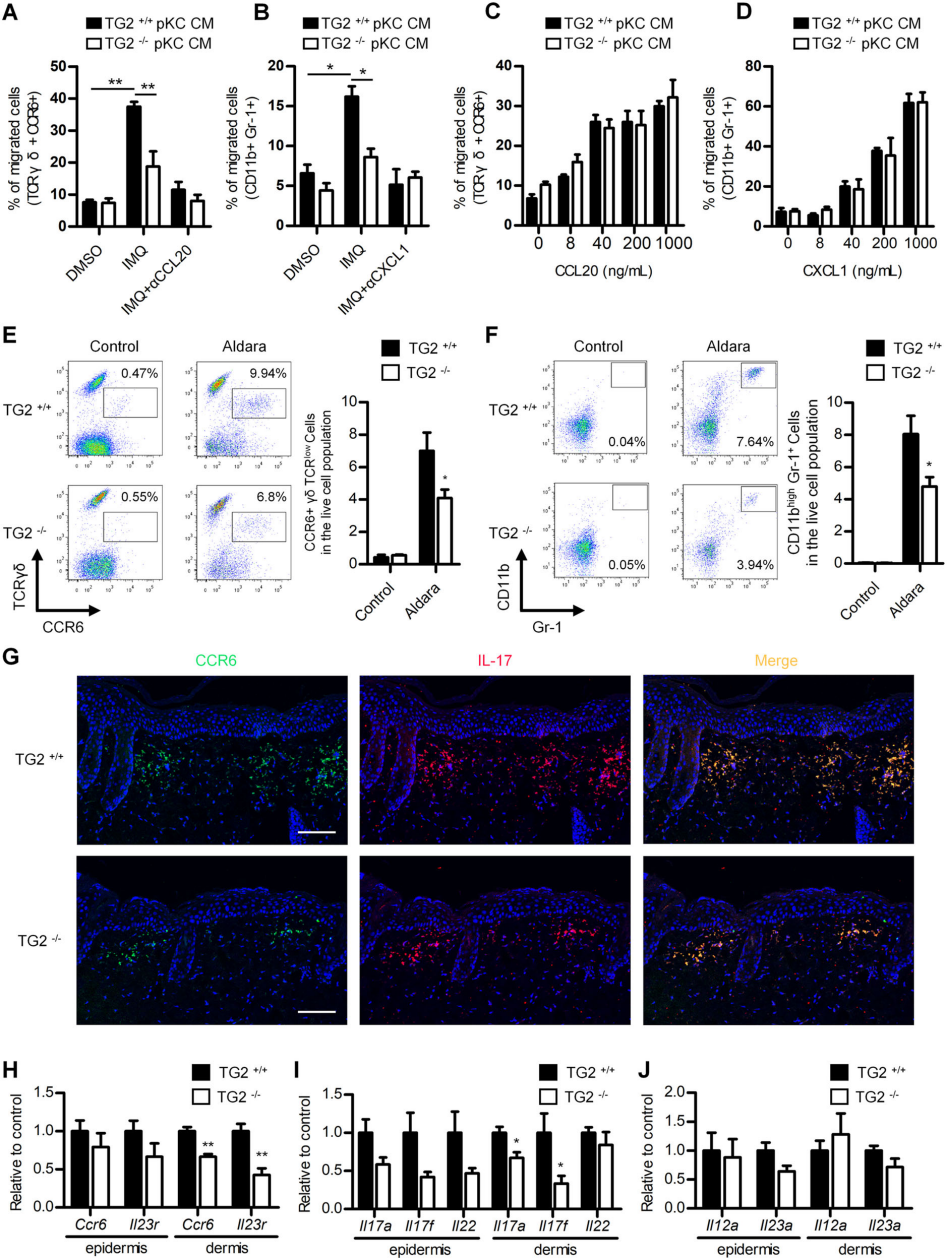
TG2-dependent chemokine production in keratinocytes is critical for IL-17-producing CCR6^+^ γδT-cell and neutrophil dermal infiltration. **a**, **b** Effect of DMSO- or IMQ-treated WT- or TG2^*−/−*^-CM on CCR6^+^ γδT-cell (**a**) and CD11b^+^ Ly-6G^+^ cell (**b**) migration according to Transwell migration assays. Flow cytometric analysis of the percentage of cells migrating toward the CM in the presence or absence of anti-CXCL1 (**a**) and anti-CCL20 (**b**) antibodies (*n* = 3/group). **c**, **d** WT and TG2^*−/−*^ mouse-derived CCR6^+^ γδT-cell (**c**) and CD11b^+^ Ly-6G^+^ cell (**d**) migration in response to recombinant mouse CCL20 and CXCL1, respectively. Data represent the mean ± SEM (*n* = 3/group). *p < 0.05; **p < 0.01. **e**, **f** Cells were isolated from the back skin of WT and TG2^*−/−*^ mice treated with IMQ for four consecutive days and analyzed by flow cytometry. Representative flow cytometric profiles of CCR6^+^ γδTCR^low^ cells (**e**) and CD11b^high^ Ly-6G^+^ cells (**f**). The percentage of cells is shown. Data represent the mean ± SEM (*n* = 9/group). **g** Skin sections were immunostained with CCR6 (green)- and IL-17 (red)-specific antibodies. Nuclei were stained with DAPI (blue). Scale bar, 100 μm. **h**–**j** Back skin was separated into epidermis and dermis and analyzed by RT-PCR for IL-17-producing γδT-cell markers (**h**) (*Ccr6* and *Il23r*), IL17 and related cytokines (**i**) (*Il17a*, *Il17f*, and *Il22*), and myeloid cell-derived cytokines (**j**) (*Il12a* and *Il23a*). Data represent the mean ± SEM (*n* = 11/group). *p < 0.05; **p < 0.01 vs. WT mice.

We then investigated the mechanism by which TG2-induced *CCL20* expression in keratinocytes promotes skin inflammation. Because CCL20 is a chemotactic factor that attracts IL17-producing CCR6^+^ T-cells, we performed Transwell migration assays with T-cells isolated from the spleens and LNs of Aldara-treated WT mice on day 4. Flow cytometric analysis showed that conditioned media (CM) from IMQ-treated WT-mKCs significantly enhanced CCR6^+^ γδT-cell migration as compared with CM from IMQ-treated TG2^*−/−*^-mKCs. Moreover, adding CCL20-neutralizing antibodies to the CM reduced CCR6^+^ γδT-cell migration (Fig. 5a). Similar results were obtained for CD11b^+^ Ly-6G^+^ neutrophils harvested from Aldara-treated mice, which exhibited enhanced migration toward the CM of WT-mKCs relative to TG2^*−/−*^-mKCs and was suppressed by CXCL1-neutralizing antibodies (Fig. 5b). To exclude the possibility of defective TG2^*−/−*^ immune-cell migration, we compared the ability of WT and TG2^*−/−*^ CCR6^+^ γδT-cells and neutrophils to migrate toward CCL20 and CXCL1, respectively. The chemokines dose-dependently increased immune-cell migration, but migration ability did not differ between the WT and TG2^*−/−*^ cells (Fig. 5c, d). These results indicate that TG2 critically regulates immune-cell migration by inducing chemokine expression in keratinocytes.

To confirm the role of TG2 in immune-cell recruitment in vivo, we prepared single-cell suspensions from the skin of WT and TG2^*−/−*^ mice treated with Aldara for 4 days. Flow cytometric analysis revealed that Aldara treatment markedly increased the number of CCR6^+^ γδTCR^low^ cells in WT skin as compared with untreated skin, whereas the effect of Aldara treatment was significantly reduced in TG2^*−/−*^ skin (Fig. 5e). A similar TG2-mediated effect was observed for CD11b^high^ Ly-6G^+^ neutrophils (Fig. 5f), whereas fluorescence confocal microscopy showed that most CCR6-expressing cells were co-stained with IL-17 in the dermis of WT skin and displayed impaired infiltration in TG2^*−/−*^ skin (Fig. 5g). Quantitative PCR confirmed that *Ccr6* and *Il23r*, as IL-17-secreting γδT-cell markers (Fig. 5h), and *Il17a* and *Il17f* (Fig. 5i) mRNA levels were significantly lower in the dermis of Aldara-treated TG2^−/−^ mice relative to levels in WT mice. Conversely, *Il12a* and *Il23a* (cytokines secreted by myeloid cells, such as DCs and macrophages) mRNA levels were comparable in the epidermis and dermis of WT and TG2^*−/−*^ mice (Fig. 5j). These results indicate that TG2 promotes IL-17-secreting γδT-cell dermal infiltration in IMQ-treated skin.

### High correlation between TG2 and psoriatic cytokine and chemokine expression in human psoriatic skin

**Fig. 6:**
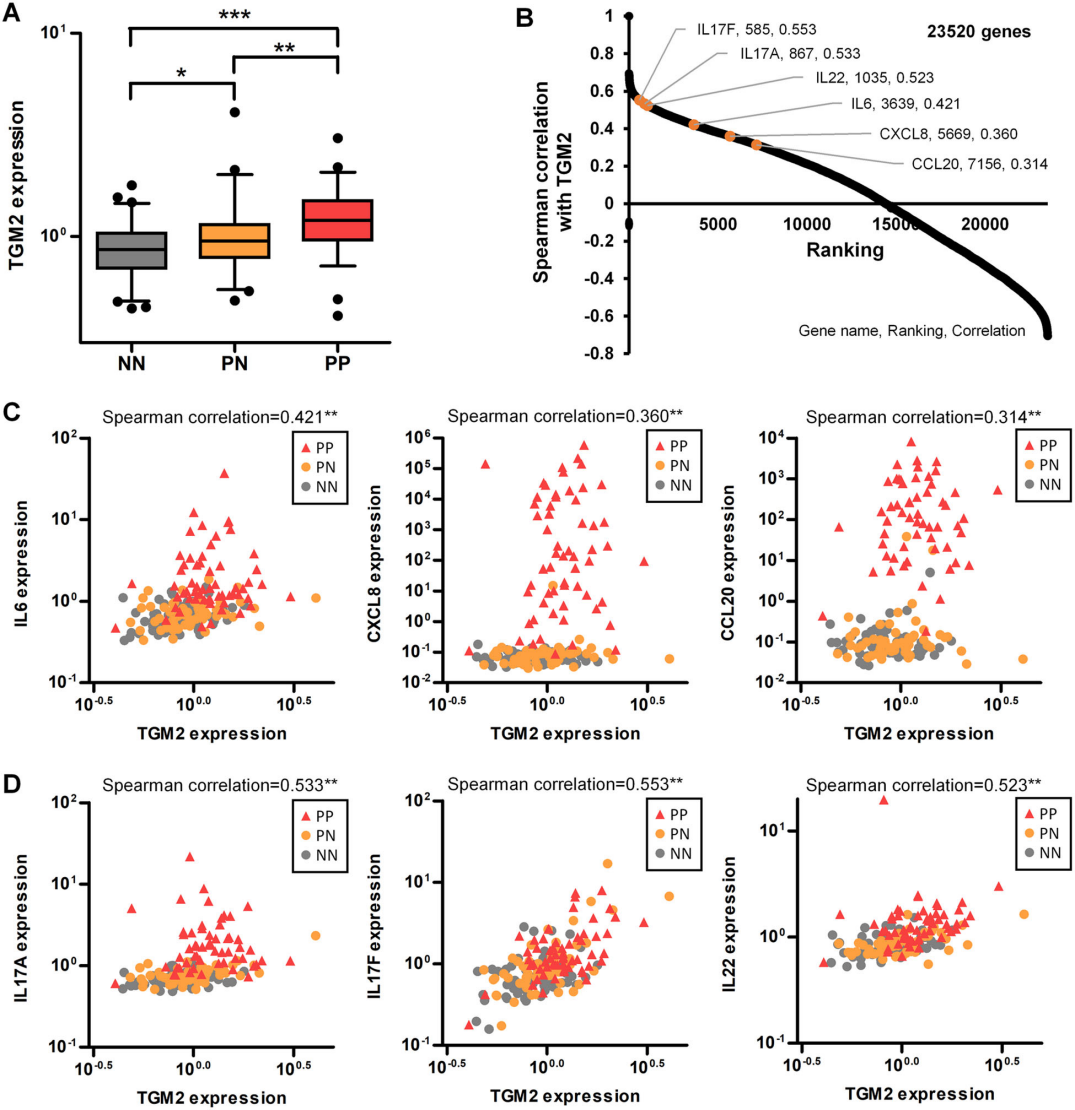
Human psoriatic skin exhibits a high correlation between TG2 and psoriatic cytokine and chemokine expression. **a**–**d** Gene-expression profile and TG2 correlation were analyzed using reported microarray data from 64 normal controls (NN) and normal skin lesions (PN) and psoriatic lesions (PP) from 58 psoriatic patients. **a** TG2 mRNA levels in NN, PN, and PP groups. *p < 0.05; **p < 0.01; ***p < 0.001. **b** Correlation between TG2 levels and 23,520 genes was analyzed by Spearman’s rank correlation. The ranking and correlation coefficient of the indicated genes are presented in the plots. Scatter plots of correlation between TG2 expression and *IL6, CXCL8*, and *CCL20* expression (**c**) and *IL17F, IL17A*, and *IL22* expression (**d**) in NN, PN, and PP groups.

We then examined whether the observed role of TG2 in the mouse model could be extended to human psoriatic inflammation by comparing TG2 and related cytokine expression levels in normal and psoriatic skin using a previously reported microarray dataset of 64 normal controls and 58 psoriatic patients^30^. As expected, *IL6*, *CXCL8*, *CCL20, IL17F*, *IL17A*, and *IL22* mRNA levels were significantly upregulated in both psoriatic lesions (PP) and the uninvolved skin of psoriatic patients (PN) as compared with normal skin (NN) (Supplementary Fig. S5). In addition, TG2 mRNA levels were significantly higher in both PP and PN than in NN. Interestingly, PP showed higher TG2 expression than that in PN (Fig. 6a), suggesting that TG2 might be implicated in lesion development. Gene-expression correlation analysis of the 23,520 genes examined in the microarray study revealed that TG2 mRNA levels showed moderate correlation with *IL6*, *CXCL8*, and *CCL20* levels (Spearman’s *R* > 0.3) and strong correlation with *IL17F*, *IL17A*, and *IL22* levels (Spearman’s *R* > 0.5) (Fig. 6b). The correlations between TG2 expression and these six genes are shown in dot plot diagrams (Fig. 6c, d). Analysis of the PP and PN (*n* = 118) datasets revealed similar correlations between TG2 and cytokine or chemokine expression. Moreover, there was a significant correlation between TG2 and *IL17F* and *IL22* expression in the PP dataset (*n* = 58) (Supplementary Table S1), suggesting that TG2 might predict disease severity. Together, these results support the role of TG2 in recruiting IL-17-producing cells in human psoriatic skin by mediating IL-6, CXCL8, and CCL20 expression.

## Discussion

The high therapeutic efficacy of IL-17-, TNF-α-, and IL-23-targeting biologics for treating psoriasis supports the central role of IL-17-producing T-cells in the development of psoriatic inflammation^31^. Keratinocyte-derived cytokines in turn amplify the IL-23/IL-17 axis by recruiting IL-17-producing T-cells to the psoriatic inflammation site, thereby forming a positive feedback loop^3^. Cytokines and chemokines secreted from intestinal and lung epithelial cells in response to pathogenic microbes or immune cell-derived cytokines increase inflammation by recruiting and activating immune cells^32,33^. In the skin, keratinocyte-derived inflammatory cytokines trigger and promote inflammation in atopic dermatitis and psoriasis^34^. We previously showed that TG2-induced IL-6 in bleomycin-treated lung epithelial cells triggers T_H_17-mediated inflammation^21^. Moreover, TG2-induced upregulation of TNF-α, IL-6, and CXCL8 levels in UV-irradiated keratinocytes leads to immune-cell infiltration and skin inflammation^22^. The present study showed that TG2-mediated *IL6*, *CXCL1*, and *CCL20* expression in IMQ-treated keratinocytes promotes γδT-cell and neutrophil recruitment, suggesting that aberrant TG2 regulation in keratinocytes might contribute to the development of psoriatic inflammation. Indeed, TG2 expression was higher in psoriatic skin than normal skin and correlated with *IL6*, *CXCL8*, and *CCL20* levels. Moreover, TG2 is not involved in T_H_17 differentiation^21^, IMQ-induced DC maturation (Supplementary Fig. S2), or γδT-cell and neutrophil migration capacity (Fig. 5c, d). Chimeric mice with TG2-deficient keratinocytes also exhibited reduced IMQ-induced inflammation, indicating that TG2 could be a promising target for suppressing psoriatic inflammation by inhibiting keratinocyte–immune-cell interactions.

IMQ activates the MyD88 signaling pathway by binding TLR7, leading to DC and macrophage maturation and the production of inflammatory cytokines, such as IL-23^35^. Moreover, type I IFN (IFN-I) secretion is elevated in plasmacytoid DCs (pDCs) of IMQ-treated mice via TLR7, resulting in psoriatic inflammation^36^. In addition to these TLR7-dependent systemic inflammatory responses, IMQ induces psoriasis-associated cytokine and chemokine expression in keratinocytes, thereby contributing to the development of local skin inflammation^12,37^; however, pDCs and IFN-I are not required for keratinocyte responses to IMQ^11,38^. Furthermore, TLR7 is not expressed in keratinocytes^37^, indicating that IMQ-induced keratinocyte responses are TLR7-independent; however, it remains unclear how IMQ stimulates cytokine expression in keratinocytes. Our results in IMQ-treated TG2^*−/−*^ and chimeric mice revealed that systemic immune responses did not differ between WT and TG2^*−/−*^ mice, whereas TG2^*−/−*^ mice exhibit significantly lower inflammatory cytokine expression levels in keratinocytes relative to WT mice, indicating that TG2 is involved in TLR7-independent keratinocyte responses to IMQ.

IMQ upregulates psoriasis-associated cytokine and chemokine expression by activating the NF-κB signaling pathway in keratinocytes^11,12^; however, the mechanism of IMQ-induced NF-κB activation is unclear. TG2 activates NF-κB signaling in various cell types, likely by cross-linking and sequestering IκB^39^. We previously showed that TG2 is required for UV-induced NF-κB activation in keratinocytes, resulting in inflammatory cytokine production^22^. In the present study, we showed that IMQ induces and activates TG2 in keratinocytes and, using CCL20-promoter-deletion constructs, demonstrated that IMQ-induced CCL20 expression mostly depends on NF-κB, and that TG2 mediates NF-κB-dependent CCL20 expression in IMQ-treated keratinocytes. Therefore, these results indicate that TG2 links IMQ to NF-κB activation and offer insight into how IMQ induces cytokine and chemokine expression in TLR7-negative keratinocytes.

Dermal γδT-cells are a major IL-17-producing cell subset that differ from skin-resistant γδT-cells, known as dendritic epidermal T-cells (DETCs). Unlike DETCs, dermal γδT-cells are characterized by intermediate TCRγδ expression and surface CCR6 expression; therefore, they are recruited to the skin in response to CCL20 produced during skin inflammation^8^. Previous studies show that dermal γδT-cells have a critical role in psoriasis pathogenesis. In both IL-23- and IMQ-induced psoriasis animal models, dermal γδT-cells mainly infiltrate the dermis and then migrate to the epidermis, which is associated with IL-17 expression levels in psoriatic lesions and highly dependent on the CCL20/CCR6 axis^9,10,40^. In the present study, our results showed that most IL-17-producing CCR6-positive cells were detected in the dermis 4 days after daily IMQ treatment, at which point *Ccr6*, *Il23r*, *Il17a*, and *Il17f* mRNA levels were significantly lower in the dermis of TG2^*−/−*^ mice relative to those in WT mice. Moreover, TG2^*−/−*^ mice exhibited impaired dermal γδT-cell trafficking, which is the first step in their skin infiltration. These results emphasize that TG2 has a critical role in the regulation of dermal γδT-cell recruitment.

In summary, we demonstrated that keratinocyte-derived TG2 mediates IL-6, CXCL8, and CCL20 expression by activating NF-κB and amplifies the interaction between keratinocytes and immune cells in psoriatic skin lesions by recruiting neutrophils and IL-17-producing γδT-cells. These findings suggest that TG2 may serve as an important target for psoriasis treatment.

## Materials and methods

### Animal

TG2^−/−^ mice^41^ were backcrossed 12 times to C57BL/6J. 10–11-week-old male mice were shaved and chemically depilated by Veet cream (Oxy-Reckitt Benckiser, Slough, UK) at 48 h before IMQ treatment. Daily topical dose (62.5 mg) of 5% IMQ cream (Aldara, 3 M Pharmaceuticals) was applied on the shaved back and the right ear for six consecutive days, as previously described^24^. The psoriasis area and severity index (PASI) were scored by two independent researchers blind to genotype. Animal experiments were approved by the Seoul National University Institutional Animal Care and Use Committee.

### Bone marrow chimeric mice

WT and TG2^−/−^ recipient mice were lethally irradiated by exposure to 950 rad of γ irradiation in two doses and T-cell-depleted BM cells (3 × 10^6^/mouse) from WT and TG2^−/−^ donor mice were transferred through the tail vein. Reconstituted mice were used in the experiments 10 weeks after the BM-cell transfer.

### Histology and immunohistofluorescence

Mouse skin tissues were fixed in 4% paraformaldehyde for 24 h, embedded in paraffin and sectioned. Sections were deparaffinized and stained with H&E for histopathological analysis. For immunohistofluorescence analysis, antigen retrieval was performed in sodium citrate buffer (10 mM Sodium Citrate, 0.05% Tween 20, pH 6.0) at 100 °C for 20 min, and blocked in 10% normal serum, 1% BSA, 0.1% Triton X-100 in PBS for 2 h at room temperature. Sections were incubated with anti-IL-17 antibody (Novus Biologicals, NBP1-72027, 1:100) and CCR6 antibody (Abnova, PAB12270, 1:250) overnight at 4 °C. After washing with PBS, sections were stained with Alexa 594 conjugated anti-Rat Ig G antibody (Invitrogen, A11007, 1:500) and Alexa 488 conjugated anti-Rabbit Ig G antibody (Invitrogen, A11008, 1:500) for 1 h at room temperature. Following PBS washing, sections were counterstained with DAPI, mounted in ProLong™ Diamond Antifade Mountant (Thermo Fisher Scientific, P36965), and analyzed under a confocal fluorescence microscope (Olympus, FV3000).

To measure keratinocyte proliferation in mouse skin tissue. 1 mg BrdU (Roche) was injected 12 h before killing. Fixed and paraffin embedded sections were deparaffinized and boiled in citrate buffer, and incubated with anti-BrdU antibody, followed by HRP-conjugated goat anti-mouse Ig G (Thermo Fisher Scientific, 31431, 1:5000)

### Cell culture

HaCaT cells were cultured in Dulbecco’s modified Eagle’s medium (Welgene, LM 001-05) containing 10% heat-inactivated fetal bovine serum (Hyclone) and 1% Penicillin–Streptomycin (Gibco, 15140122). TG2-deficient stable HaCaT-cell line was established by transduction of shTG2 and shCON expressing lentiviruses. To obtain the lentivirus, shRNA lentiviral construct targeting to TGM2 (Sigma-Aldrich, TRCN0000000239) or non-target shRNA was co-transfected with lentivirus packaging plasmids into HEK293FT-cells using lipofectamine 3000 reagent (Thermo Fisher Scientific, L3000008). After the transduction, cells were selected with 1 μg/ml of puromycin (InvivoGen, ant-pr-1) for 1 week. All cell lines were regularly tested to exclude mycoplasma contamination.

Primary mouse epidermal keratinocytes (mKC) were prepared from neonatal skin as described previously^22^. mKCs were cultured in keratinocyte proliferation media (1:1 mixture of KGM-Gold and calcium free KGM-Gold kit media, Lonza) and used at passage number 1.

The primary human keratinocytes (hKC) were kindly provided by Dr. JH Chung (Seoul National University). hKC were cultured in primary keratinocyte media (KGM-Gold BulletKit, Lonza, 192060). TG2-deficient hKCs were generated by transduction of shTG2 lentivirus. In brief, when the cells were grown to 30% confluency, cells were treated with lentivirus solution and 8 μg/ml polybrene overnight. Next day, cells were washed and the culture media replaced with fresh primary keratinocyte media. 48 h after the media change, transduced hKCs were used in experiments.

### Real-time PCR

Total RNA from cells was prepared using Trizol reagent (Invitrogen) according to the manufacturer’s instructions. Mouse back skin tissues were separated to dermis and epidermis by incubating in dispase solution (5 mg/ml in DMEM with 10% FBS) at 37 °C for 2 h, and homogenized in TRIzol reagent (Invitrogen) using a homogenizer (Ultra-Turrax Dispenser, IKA) and total RNA was extracted according to manufacturer’s instructions. 1 µg of total RNA was reverse transcribed to cDNA with oligo dT and Superscript II (Invitrogen). Real-time PCR was performed using CFX96 real-time system (Bio-Rad) and SYBR qPCR master mix (Kapa biosystems). Real-time PCR primer sequences are listed in Supplementary Table S2.

### Beads-based cytokine immunoassays

Secreted human or mouse IL-6, and CXCL8 protein concentrations were measured in culture supernatants using Cytometric Bead Array (BD Biosciences) according to manufacturer’s instructions. Human or mouse CCL20 protein concentrations in conditioned media were measured using LEGENDplex CCL20 capture beads and protein standards (Biolegend) as recommended by manufacturer’s instruction. Secreted protein concentration was normalized to total cellular protein level determined using BCA assay.

### Western blot

Sample preparation and SDS-PAGE were performed as previously described^21^. The following primary antibodies were used for immunoblotting: anti-β-actin (Sigma-Aldrich, A5441) and anti-TG2^42^.

### In situ transglutaminase (TG) assay

In situ TG activity assay was performed as previously described^43^. Flowing IMQ treatment, HaCaT cells were incubated with 1 mM EZ-link Pentylamine-Biotin (BP) (Thermo Fisher Scientific, 21345) as an amine donor, which is incorporated into glutamine residues of TG substrate proteins, in serum-free DMEM for 1 h. Total cell protein extracts were prepared and the BP-incorporated TG substrates were visualized by western blotting using HRP-conjugated streptavidin (Thermo Fisher Scientific, 21126) and the ECL Detection Reagent. The signal on Western blot was quantified using ImageJ software.

### Luciferase assay

HaCaT cells were plated at 6 × 10^3^ cells per well of 48-well plate 48 h before transfection. To measure NF-κB activity, 3× κB-luc (300 ng) and pRL-TK (100 ng) were transfected into cells using lipofectamine 3000 (Invitrogen). To perform the CCL20-promoter assay, CCL20-promoter mutant constructs (300 ng) and pRL-TK (100 ng) were transfected. CCL20-promoter mutant constructs were kindly provided by Dr. Fang Liao^29^. 24 h after transfection, cells were treated with IMQ (200 μM) for 6 h and luciferase activity were measured using the Dual Luciferase Assay Kit (Promega) following manufacturer’s instructions.

### Migration assay

WT and TG2^−/−^ mice were topically applied with Aldara cream for 4 days, and T-cells and granulocytes were purified from the LNs and spleens. T-cells were isolated using pan T-cell isolation kit II (Miltenyi Biotec, 130-095-130) according to manufacturer’s instructions. To isolate granulocytes, cells from LNs and spleen were labeled with biotinylated GR-1 antibody (BioLengend, 108403) and separated by using Anti-Biotin MicroBeads (Miltenyi Biotec, 130-090-485) and MS Columns (Miltenyi Biotec, 130-042-201) according to manufacturer’s instructions. Migration assays were performed using 24-well Transwell plates with a 3.0 µm polycarbonate membrane (SPL, 35124). Recombinant mouse CCL20 (BioLegend, 582304), CXCL1 (BioLegend, 573704), or conditioned media collected from WT or TG2^−/−^ mouse primary keratinocytes treated with IMQ for 24 h with or without 1 μg/ml of anti-CCL20 (R&D Systems, MAB7601) or anti-CXCL1 (R&D Systems, MAB453) neutralizing antibody were placed on the lower chamber. Isolated T-cells or Gr-1+ granulocytes were preincubated in RPMI1640 (Welgene, LM 011-01) containing 1% fatty acid–free bovine serum albumin (Sigma-Aldrich, A8806), 2 mM glutamine (Thermo Fisher Scientific, 25030081), and 20 mM HEPES (Thermo Fisher Scientific, 15630106) for 30 min at 37 °C 5% CO_2_, then 1 × 10^6^ cells were placed in upper chamber in a 100 μl volume for 6 h. Migrated neutrophils (CD11b^+^ Ly-6G^+^) and CCR6 + γδT-cells (TCR γδ^+^ CCR6^+^) were counted by flow cytometry using Precision Count Beads (BioLengend, 424902) following manufacturer’s instructions. The migration percentage was calculated by dividing the number of migrated cells by the number of cells placed in upper chamber.

### Flow cytometry

Mouse back skin was minced using scissors into small pieces (<2 mm) and incubated at 37 °C for 90 min in RPMI1640 containing 5% fetal bovine serum, 300 μg/ml Liberase TM (Sigma-Aldrich, 5401119001), 50 U/ml DNAse I (Sigma-Aldrich, D4263). Following enzyme digestion, single-cell suspensions were prepared by filtering through 70 μm nylon strainers. The following primary antibodies were used for flow cytometry: BV785-anti-mouse/human CD11b (#101243), Alexa Fluor 647-anti-mouse Ly-6G (#127610), and PE-anti-mouse CCR6 (#129803) were purchased from BioLegend. BV421-anti-Mouse γδTCR (#562892) was purchased from BD bioscience. Flow cytometry was performed on LSR Fortessa using FACS DIVA software (BD bioscience), and all data were analyzed using Flowjo 7.6.

### Gene-expression correlation analysis

A microarray dataset downloaded from the Gene Expression Omnibus (GEO) was analyzed, accession number GSE13355. The data were previously analyzed in human skin biopsy samples obtained from the buttock of 64 control individuals, and from psoriasis plaques and normal skin region of 58 psoriatic subjects^30^. Gene-expression correlation was evaluated by calculating Spearman’s rank-order correlation coefficient values using R 3.6.1 and SPSS 25 (IBM).

### Statistical analysis

Three replicate independent experiments in triplicate were performed for the in vitro study. Data normality was assessed using the Shapiro–Wilk test. The Levene test was used to check the homogeneity of variances. One‐way analysis of variance (ANOVA) with LSD post-hoc test was used to evaluate mean differences among experimental groups. Two‐way repeated ANOVA analysis was performed to compare the day‐dependent increase of the PASI score among groups. The Tukey’s honestly significant difference (HSD) test was used for multiple comparison. Null hypotheses were rejected at significance levels of *p* < 0.05. The results were expressed as mean ± SEM and analyzed using SPSS 25 (IBM).

## Supplementary information

Supplementary Fig. S1

Supplementary Fig. S2

Supplementary Fig. S3

Supplementary Fig. S4

Supplementary Fig. S5

Supplementary Table. S1

Supplementary Table. S2

Supplementary Figure Legends
