# Supplementary figures and images for: Keratinocyte transglutaminase 2 promotes CCR6+ γδT-cell recruitment by upregulating CCL20 in psoriatic inflammation
Source: Cell Death Dis. 2020 Apr 30;11(4):301. doi: 10.1038/s41419-020-2495-z (PMC7193648; doi:10.1038/s41419-020-2495-z)

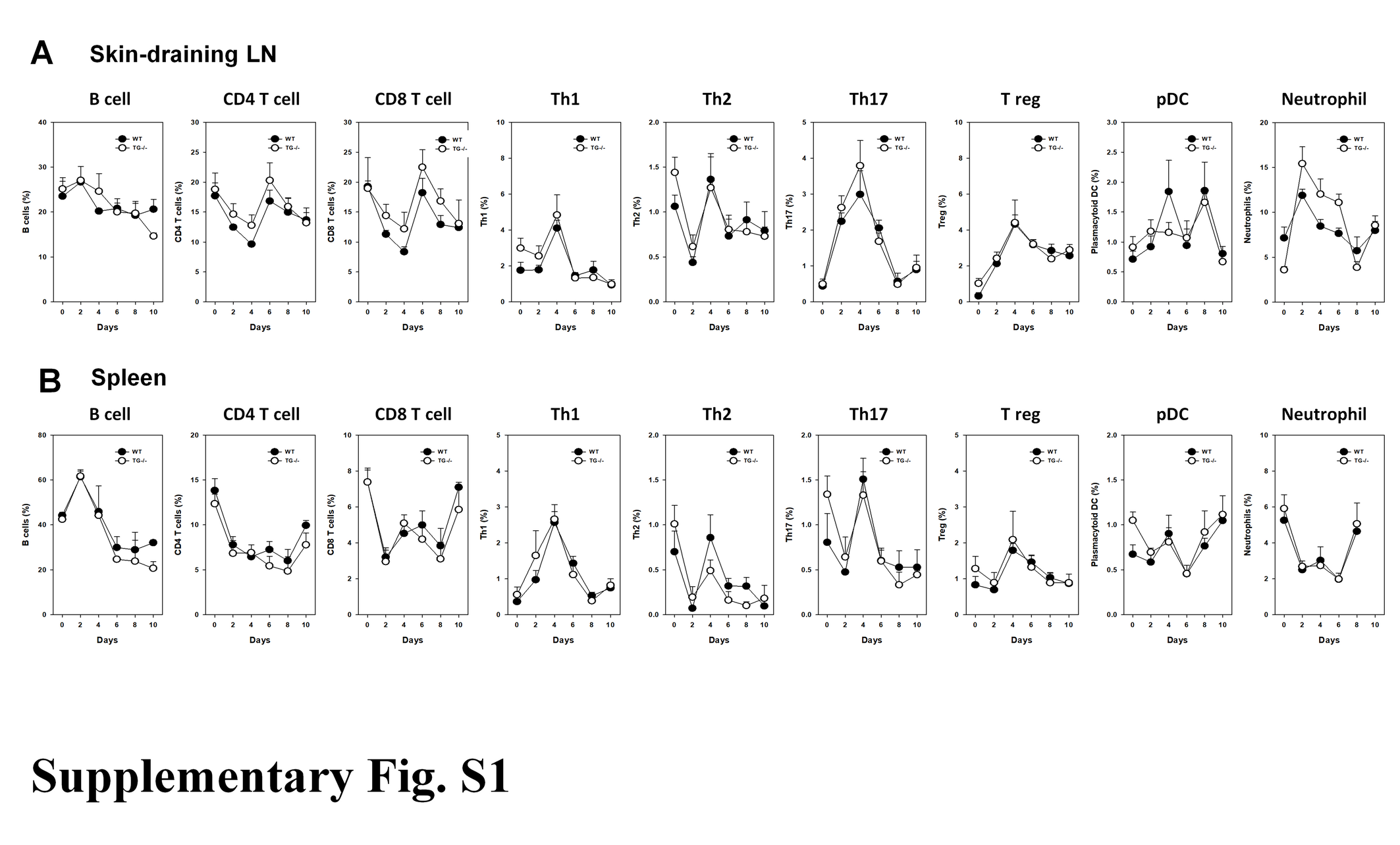

Supplement: Supplementary file 1 — Supplementary Fig. S1 [file 41419_2020_2495_MOESM1_ESM.tif]

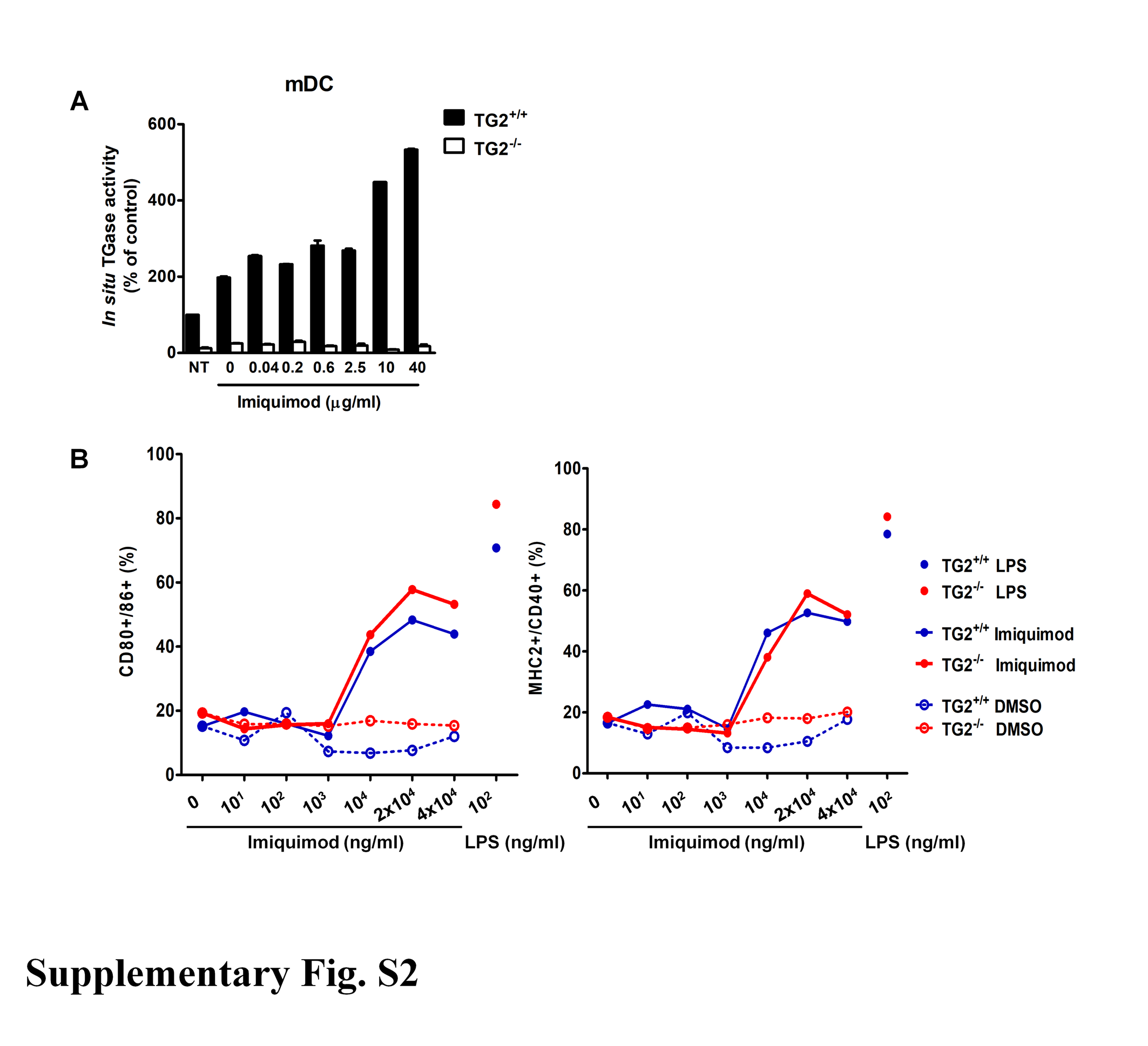

Supplement: Supplementary file 2 — Supplementary Fig. S2 [file 41419_2020_2495_MOESM2_ESM.tif]

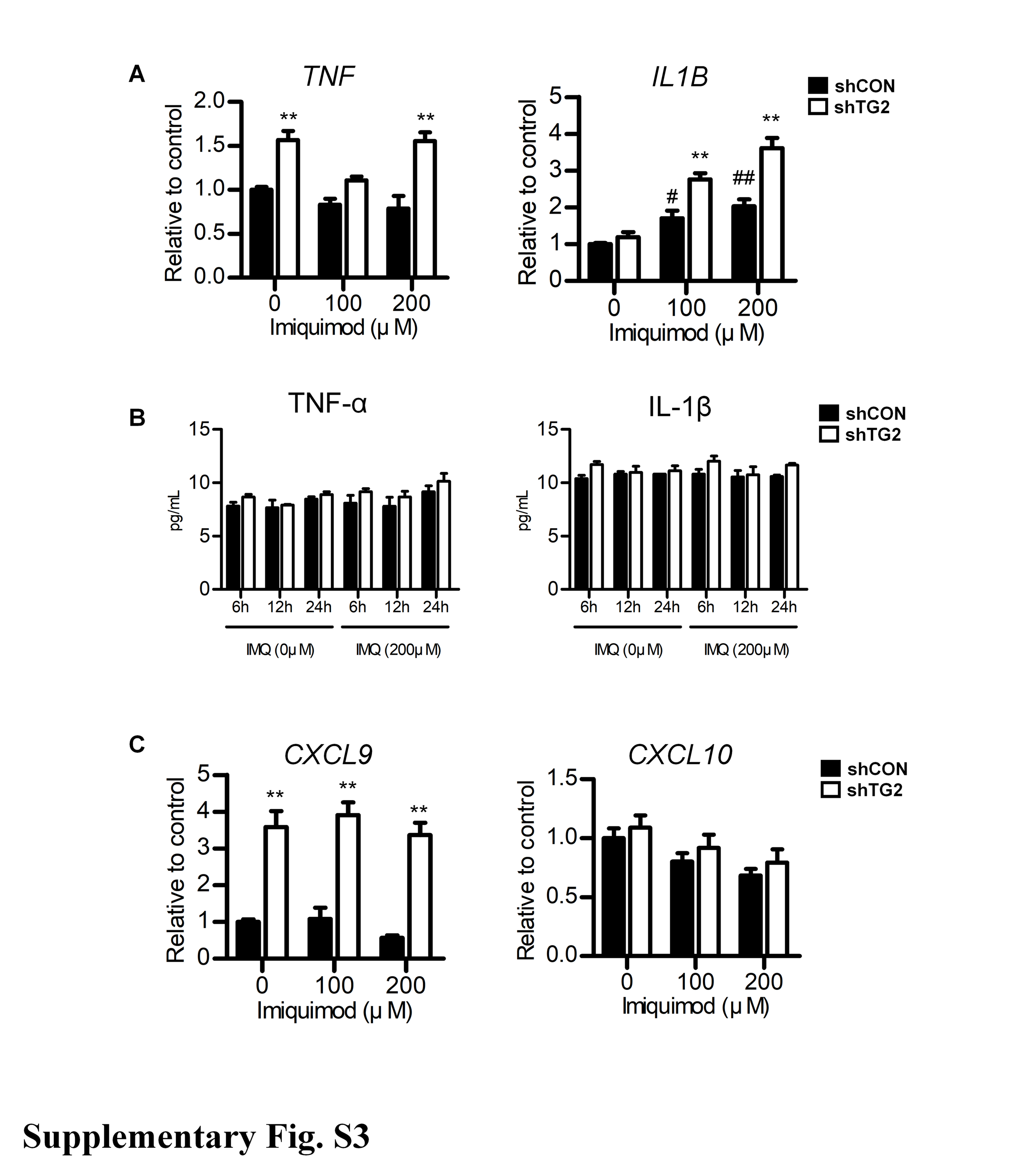

Supplement: Supplementary file 3 — Supplementary Fig. S3 [file 41419_2020_2495_MOESM3_ESM.tif]

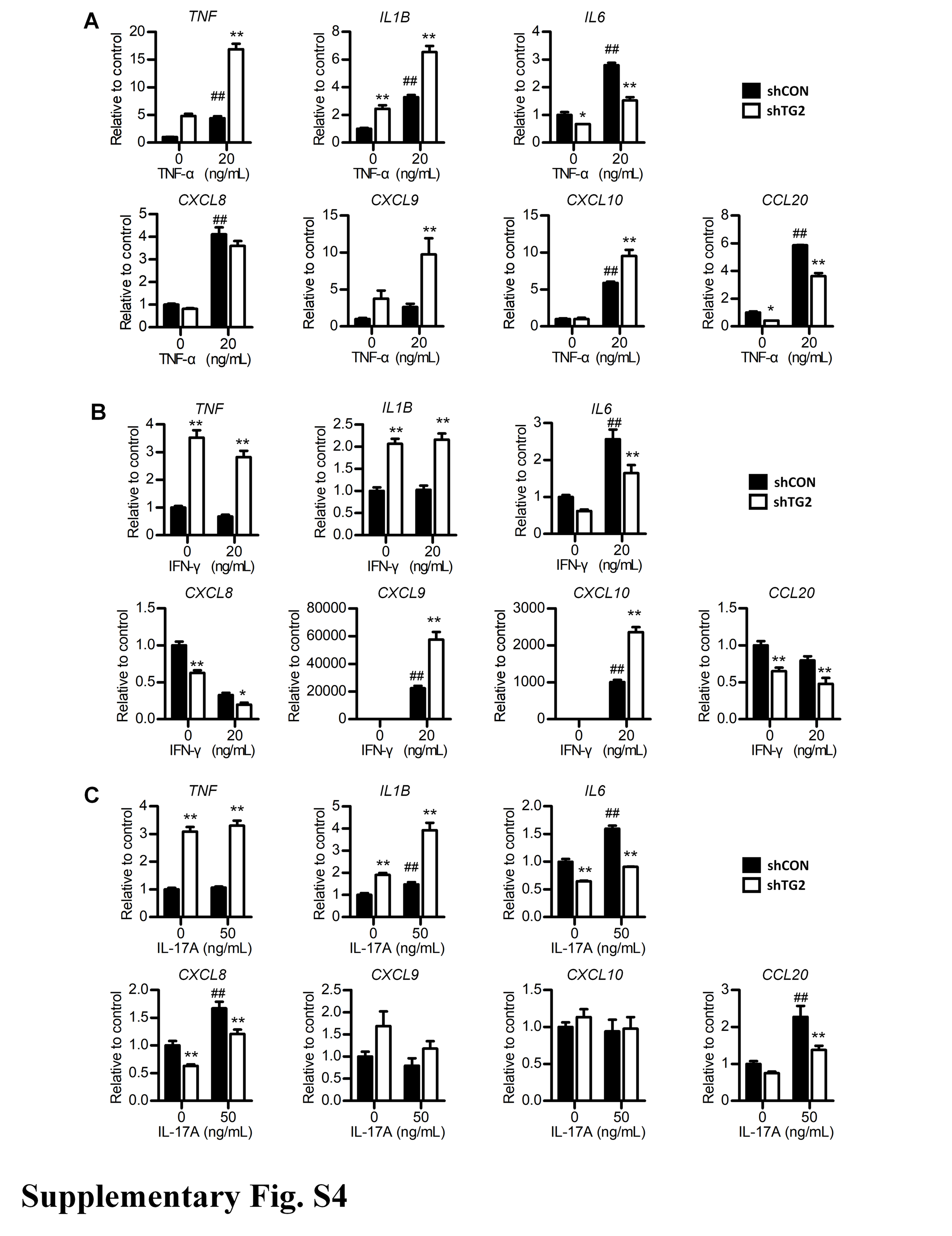

Supplement: Supplementary file 4 — Supplementary Fig. S4 [file 41419_2020_2495_MOESM4_ESM.tif]

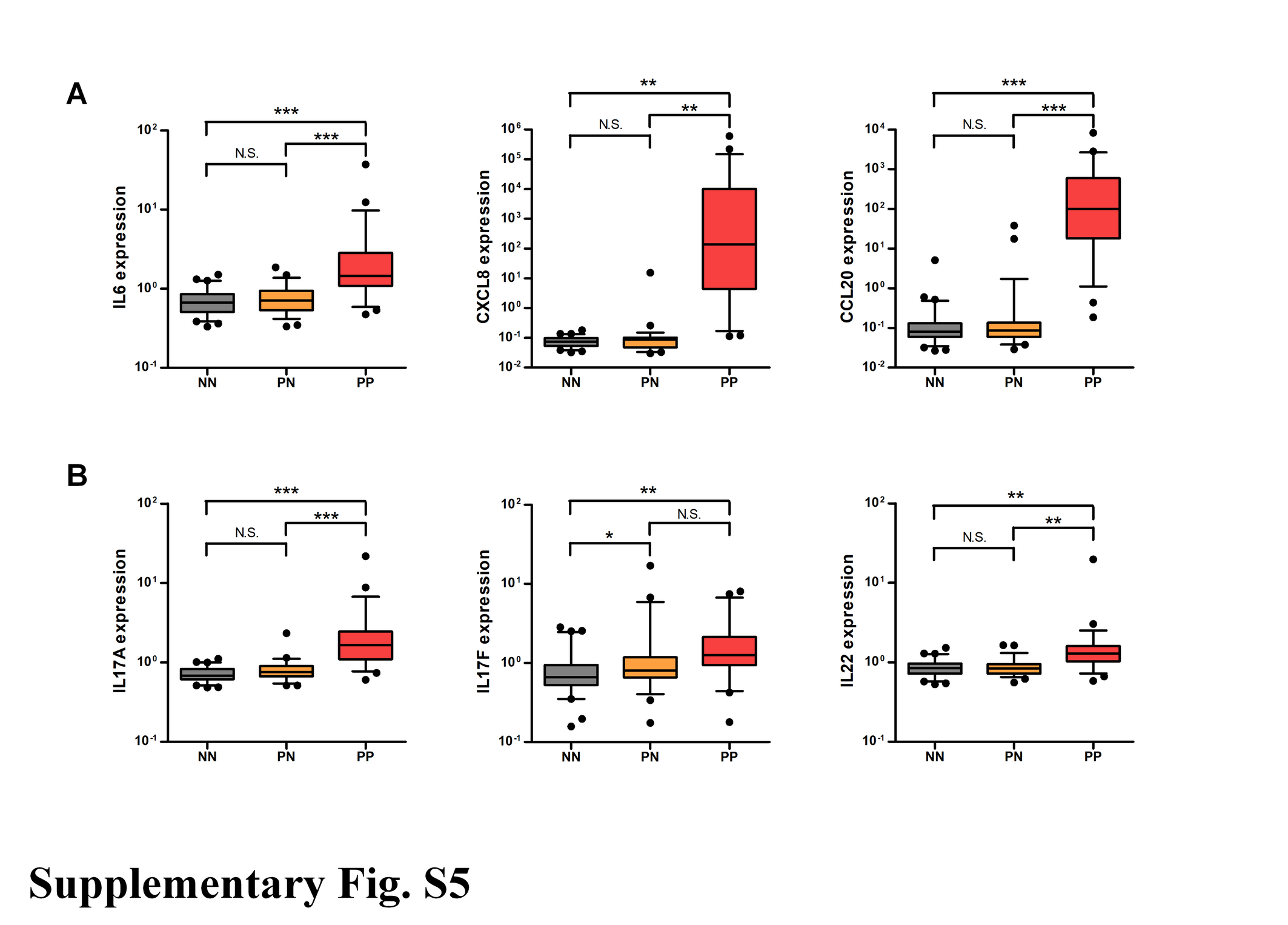

Supplement: Supplementary file 5 — Supplementary Fig. S5 [file 41419_2020_2495_MOESM5_ESM.tif]
