# Supplementary material for: Keratinocyte transglutaminase 2 promotes CCR6+ γδT-cell recruitment by upregulating CCL20 in psoriatic inflammation
Source: Cell Death Dis. 2020 Apr 30;11(4):301. doi: 10.1038/s41419-020-2495-z (PMC7193648; doi:10.1038/s41419-020-2495-z)
Supplement: Supplementary file 6 — Supplementary Table. S1 [file 41419_2020_2495_MOESM6_ESM.docx]

**Supplementary Table S1. Correlation Coefficient values between mRNA levels of TG2, and psoriatic cytokines and chemokines by combining sample group.**

|  |  | TG2 | IL6 | CXCL8 | CCL20 | IL17A | IL17F | IL22 |
| --- | --- | --- | --- | --- | --- | --- | --- | --- |
| Total samples (NN, PN, and PP, n=180) | Correlation Coefficient | 1.000 | .421^**^ | .360^**^ | .314^**^ | .533^**^ | .553^**^ | .523^**^ |
|  | p-value (2-tailed) |  | .000 | .000 | .000 | .000 | .000 | .000 |
| Psoriasis samples (PN and PP, n=116) | Correlation Coefficient | 1.000 | .363^**^ | .354^**^ | .321^**^ | .472^**^ | .571^**^ | .522^**^ |
|  | p-value (2-tailed) |  | .000 | .000 | .000 | .000 | .000 | .000 |
| Psoriatic lesion (PP, n=58) | Correlation Coefficient | 1.000 | .063 | .120 | .034 | .054 | .404^**^ | .338^**^ |
|  | p-value (2-tailed) |  | .637 | .371 | .802 | .689 | .002 | .009 |

*, **. Correlation is significant at the 0.05 and 0.01 level, respectively (2-tailed).
