# Supplementary material for: Keratinocyte transglutaminase 2 promotes CCR6+ γδT-cell recruitment by upregulating CCL20 in psoriatic inflammation
Source: Cell Death Dis. 2020 Apr 30;11(4):301. doi: 10.1038/s41419-020-2495-z (PMC7193648; doi:10.1038/s41419-020-2495-z)
Supplement: Supplementary file 7 — Supplementary Table. S2 [file 41419_2020_2495_MOESM7_ESM.docx]

**Supplementary Table S2. Primer sequences used in qRT-PCR reactions.**

| Species | Gene | Forward | Reverse |
| --- | --- | --- | --- |
| Human | *CXCL10* | TACAGCAGAGGAACCTCCAGTCTC | AGAGAGAGGTACTCCTTGAATGCC |
|  | *CXCL9* | GGTTCTGATTGGAGTGCAAGGAAC | AGGTGGATAGTCCCTTGGTTGG |
|  | *IL1B* | GAACTGAAAGCTCTCCACCTCCAG | AAAGGACATGGAGAACACCACTTG |
|  | *TNF* | AGCCTGTAGCCCATGTTGTAGC | ATCTCTCAGCTCCACGCCATTG |
|  | *IL6* | CTATGAACTCCTTCTCCACAAGCG | GGGCGGCTACATCTTTGGAATC |
|  | *CCL20* | GAGTTTGCTCCTGGCTGCTTTG | AGCAGTCAAAGTTGCTTGCTTCTG |
|  | *CXCL8* | TCTGCAGCTCTGTGTGAAGGTG | TGTGGTCCACTCTCAATCACTCTC |
|  | *ACTB* | ACTGAGCATCGAGTCCCTGATTTC | TTGTGAAGGCAGCCAGTTCTGC |
| Mouse | *Tnf* | TTCCCAAATGGCCTCCCTCTCATC | TCCTCCACTTGGTGGTTTGCTAC |
|  | *Il1b* | TGCCACCTTTTGACAGTGATG | TGATGTGCTGCTGCGAGATT |
|  | *Il6* | TGATTGTATGAACAACGATGATGC | GGACTCTGGCTTTGTCTTTCTTGT |
|  | *Cxcl9* | TCTGCCATGAAGTCCGCTGTTC | TTCCTCGAACTCCACACTGCTC |
|  | *Cxcl10* | GCCCACGTGTTGAGATCATTGC | CGTGGCTTCACTCCAGTTAAGGAG |
|  | *Ccl20* | TCCTTGCTTTGGCATGGGTACTG | GCAACAGTCGTAGTTGCTTGCTTC |
|  | *Cxcl1* | CTGGGATTCACCTCAAGAACATC | CAGGGTCAAGGCAAGCCTC |
|  | *Gapdh* | AACTTTGGCATTGTGGAAGG | ACACATTGGGGGTAGGAACA |
|  | *Il12a* | ACGGGACCAAACCAGCACATTG | AGCCAGGCAACTCTCGTTCTTG |
|  | *Il23a* | TCCAGTGTGAAGATGGTTGTGAC | TTGCAAGCAGAACTGGCTGTTG |
|  | *Ccr6* | TTGCTTCACCTCTGCTCTCCCAAC | AAGGGAAGTGGGCAGTTCAACC |
|  | *Il23r* | GAGGACATCCTGCTTCAGGTAATC | GCAACATGATGGCCAAGAAGACC |
|  | *Il17a* | TCCAGGGAGAGCTTCATCTGTGTC | TTGGACACGCTGAGCTTTGAGG |
|  | *Il17f* | TCCCACGTGAATTCCAGAACCG | GGTCTCGAGTGATGTTGTAATCCC |
|  | *Il22* | AACTGTTCCGAGGAGTCAATGC | TCTTCCAGGGTGAAGTTGAGCAC |
