# Supplementary material for: Keratinocyte transglutaminase 2 promotes CCR6+ γδT-cell recruitment by upregulating CCL20 in psoriatic inflammation
Source: Cell Death Dis. 2020 Apr 30;11(4):301. doi: 10.1038/s41419-020-2495-z (PMC7193648; doi:10.1038/s41419-020-2495-z)
Supplement: Supplementary file 8 — Supplementary Figure Legends [file 41419_2020_2495_MOESM8_ESM.docx]

**Supplementary Figure Legends**

**Fig. S1.** **Comparison of IMQ-induced activation of immune cells between WT and TG2^-/-^ mice.** (a, b) Aldara cream was topically applied to the shaved back skin of wild-type and TG2^-/-^ mice for six consecutive days. Cells were isolated from the lymph nodes (a) and spleens (b) of IMQ-treated WT and TG2^-/-^ mice and analyzed by flow cytometry using cell-specific markers for each immune cell.

**Fig. S2. TG2 is not involved in the differentiation of DC *in vitro*.** (a) Bone marrow cells from WT and TG2^-/-^ mice were differentiated into immature DC for 6 days using GM-CSF and IL-4. Cells were treated with various concentration of IMQ. *In situ* TG activity were measured in immature DC isolated from WT and TG2^-/-^ mice. (b) DC maturation was determined by the percentage of CD80 and CD86, or MHC class II and CD40 double positive cell using flow cytometry.

**Fig. S3. IMQ-induced expression of psoriasis-associated cytokine in control and TG2-knockdowned HaCaT cells.** (a - c) HaCaT cells were stably transfected with control and TG2-specific shRNA and treated with IMQ. mRNA levels of *TNF*, *IL1b* (a), *CXCL9* and *CXCL10* (c) were determined by real-time PCR. Protein levels of TNF-a and IL1b were determined by a multiplex cytometric bead array (b). Data represent mean ± SEM (n = 3/group). *, *p* < 0.05; **, *p* < 0.01 compared with shCON HaCaT cell. #, *p* < 0.05; ##, *p* < 0.01 compared with DMSO control.

**Fig. S4. TNF-α, IL-17A, and IFN-γ-induced expression of psoriasis-associated cytokine in control and shTG2 HaCaT cells.** (a - c) HaCaT cells were stably transfected with control and TG2-specific shRNA and were treated with TNF-α (a), IFN-γ (b), and IL-17A (c), then the mRNA levels of psoriasis-associated pro-inflammatory cytokine and chemokine were determined by real-time PCR. Data represent mean ± SEM (n = 3/group). *, *p* < 0.05; **, *p* < 0.01 compared with shCON HaCaT cell. #, *p* < 0.05; ##, *p* < 0.01 compared with DMSO control.

**Fig. S5. Comparison of mRNA levels for TG2-related cytokines and chemokines between normal and psoriasis patient skin.** Expression levels of IL6, CXCL8, and CCL20 (a); IL17A, IL17F and IL20 (b) were compared between 64 normal controls (NN), normal skin lesions (PN) and psoriatic lesions (PP) of 58 psoriatic patients from reported microarray data. N.S.; not significant. *, *p* < 0.05; **, *p* < 0.01; ***, *p* < 0.001.

**Table S1. Correlation Coefficient values between mRNA levels of TG2, and psoriatic cytokines and chemokines by combining sample group.**

**TableS2. Primer sequences used in qRT-PCR reactions.**
